# Cognitive reappraisal mediated the relationship between childhood emotional neglect and suicidality in depressed adolescents and young adults with NSSI behavior

**DOI:** 10.3389/fpsyt.2026.1792299

**Published:** 2026-05-13

**Authors:** Keliang Pan, Tingyu Hu, Guojian Yan, Guocheng Zhao, Yu Wang, Wanhong Peng, Zheyu Liu, Shiya Zhang, Lan Hu

**Affiliations:** The Fourth People’s Hospital of Chengdu, Chengdu, China

**Keywords:** adolescent depression, childhood emotional neglect/abuse, cognitive reappraisal, non-suicidal self-injury (NSSI), suicidal ideation

## Abstract

**Background:**

Depressed adolescents and young adults with non-suicidal self-injury (NSSI) represent a high-risk group for suicide. Childhood trauma is a well-established distal risk factor for suicidality, but the specific associations between trauma subtypes and suicidal ideation, and the underlying psychological mechanisms—particularly the roles of emotion regulation strategies—remain unexplored in this comorbid population.

**Methods:**

A cross-sectional study involving 120 depressed adolescents and young adults (aged 11-22) with NSSI was conducted. Participants completed the Childhood Trauma Questionnaire-Short Form (CTQ-SF), the Beck Scale for Suicidal Ideation (BSS), and the Emotion Regulation Questionnaire (ERQ). Pearson correlation and stepwise linear regression analyses were employed to examine relationships between childhood trauma subtypes (emotional abuse, physical abuse, sexual abuse, emotional neglect, physical neglect), emotion regulation strategies (cognitive reappraisal, expressive suppression), and current suicidal ideation. Mediation analysis with 5,000 bootstrap samples was performed to test whether cognitive reappraisal and expressive suppression mediated the link between significant trauma predictor(s) and suicidal ideation.

**Results:**

Emotional neglect (68.3%), emotional abuse (55.8%), and physical neglect (52.5%) were the most prevalent trauma subtypes. Emotional abuse, emotional neglect, and physical neglect were positively correlated with suicidal ideation, while cognitive reappraisal was negatively correlated with both emotional neglect and suicidal ideation. Stepwise linear regression identified emotional neglect as a significant predictor of both lower cognitive reappraisal and higher suicidal ideation. Crucially, cognitive reappraisal partially mediated the relationship between childhood emotional neglect and current suicidal ideation, accounting for 16.9% of the total effect. No significant associations were found for expressive suppression.

**Conclusion:**

This study highlights the predominance of emotional maltreatment in depressed youth with NSSI and identifies a specific psychological pathway: childhood emotional neglect contributes to increased suicidal ideation, partially through the impairment of cognitive reappraisal. These findings underscore the clinical importance of assessing emotional neglect and targeting cognitive reappraisal skills in therapeutic interventions aimed at reducing suicide risk in this vulnerable population.

## 1 Introduction

Nonsuicidal self-injury (NSSI) has emerged as a significant public health challenge, particularly among adolescents and young adults diagnosed with depression (1, 2). The clinical complexity of this population is heightened by the behavioral overlap between NSSI and suicidal acts, as both involve self-directed bodily harm. This diagnostic ambiguity is further compounded by robust evidence indicating that NSSI elevates an individual’s subsequent risk for suicide, especially when its frequency reaches a clinical threshold (3, 4). Given that suicide represents the most severe potential outcome of NSSI, elucidating the risk factors and underlying mechanisms of suicide in depressed adolescents and young adults with NSSI is of critical clinical importance.

The stress-diathesis model provides a valuable theoretical framework, conceptualizing suicide as the outcome of an interaction between state-dependent proximal stressors and a trait-like distal vulnerability (5). Childhood trauma represents one of the most well-established distal risk factors for the development of suicidal thoughts and behaviors. For the purpose of this study, childhood trauma is defined by the World Health Organization (WHO) as “all types of physical and/or emotional ill-treatment, sexual abuse, neglect, negligence and commercial or other exploitation, which results in actual or potential harm to the child’s health, survival, development or dignity," occurring before the age of 18 (6). (6). Adolescents with affective disorders and comorbid NSSI consistently report higher levels of childhood trauma compared to their non-NSSI counterparts (7, 8). Furthermore, childhood trauma has been robustly linked to increased suicide risk across diverse populations (9–11). For instance, an epidemiological survey of Chinese adolescents found that those exposed to any form of childhood maltreatment were 1.74 times more likely to report suicidal ideation (10). Of particular note is the fact that young individuals with a history of childhood trauma seldom report exposure to an isolated traumatic event; instead, they are more likely to have endured multiple forms of traumatic experiences over time (12). However, to our knowledge, the specific association between childhood trauma subtypes and suicide risk—and the psychological mechanisms underlying this link—remains unexplored within the high-risk population of depressed adolescents and young adults with NSSI.

A key pathway through which childhood trauma may confer suicide risk is via emotion dysregulation. Childhood trauma is a well-documented risk factor for impairments in emotion regulation (12, 13), often manifesting as maladaptive use of specific regulatory strategies. Among these, cognitive reappraisal and expressive suppression are two foundational strategies distinguished by their temporal application within the emotional response sequence (14). Cognitive reappraisal involves the cognitive reframing of an emotionally evocative situation to modify its impact, while expressive suppression entails the inhibition of ongoing emotional expression. Generally, adaptive use of reappraisal is associated with more positive emotional and interpersonal outcomes, whereas reliance on suppression is linked to poorer psychological adjustment (14).

Evidence suggests that individuals with a history of childhood maltreatment not only favor expressive suppression but also exhibit a diminished capacity to employ cognitive reappraisal to mitigate negative affect (15). In adolescent samples, mediation studies have established that both strategies are influenced by childhood trauma and, in turn, mediate its relationship with various forms of psychopathology, including anxiety and obsessive-compulsive disorder (16, 17). Critically, while suppression appears to exacerbate the negative psychological sequelae of trauma, reappraisal may buffer its detrimental effects (18).

Within suicide-specific research, emotion regulation strategies have been identified as significant mediators. For example, one study found that access to such strategies mediated the link between childhood maltreatment and 18-mongth suicidal ideation in psychiatrically hospitalized adolescents (19). Another study involving males with heroin use disorder reported an indirect effect of childhood trauma on suicidal ideation via deficits in cognitive reappraisal (20). Despite this growing evidence, significant gaps remain. No study has yet examined the distinct mediating roles of cognitive reappraisal and expressive suppression in the relationship between childhood trauma and suicidal ideation specifically among depressed adolescents and young adults with NSSI. Furthermore, it remains unclear whether these regulatory strategies function differently across specific subtypes of childhood trauma in their association with suicidal outcomes.

Therefore, the present study aims to address these gaps by focusing on depressed adolescents and young adults with NSSI. Specifically, our objectives are threefold:

1. To present the distribution characteristics of childhood trauma subtypes among depressed adolescents and young adults with NSSI.
2. To examine the relationships between childhood trauma subtypes and current suicidal ideation, as well as two emotion regulation strategies (cognitive reappraisal and expressive suppression).
3. To explore the potential mediating roles of cognitive reappraisal and expressive suppression in the links between different childhood trauma subtypes and suicidal ideation.

## 2 Methods

### 2.1 Participants

The study protocol adhered to the principles outlined in the Declaration of Helsinki and received approval from the Ethics Committee of the Chengdu Fourth People’s Hospital. All participants provided written informed consent after receiving a detailed explanation of the study procedures. For participants under 18 years of age, consent was also obtained from their legal guardians. The inclusion criteria for adolescents and young adults with depression and NSSI behavior were as follows: (1) age between 10 and 25 years; (2) either meeting the DSM-5 diagnostic criteria for major depressive disorder or presenting with clinically significant depressive symptoms; and (3) a history of at least one episode of NSSI in the past year. NSSI was defined according to established criteria as deliberate, self-inflicted damage to body tissue without suicidal intent and for purposes not socially sanctioned (21). Psychiatric diagnoses and NSSI assessments were conducted by a qualified psychiatrist. To enhance the reliability of NSSI identification, the Ottawa Self-Injury Inventory (OSI) (22) was additionally employed as a self-report measure to assess NSSI behaviors. Specifically, participants were required to report at least one or more non-suicidal self-injurious acts within the past month, six months, or year, as assessed by the OSI. Exclusion criteria for all participants included: (1) current or past severe medical or neurological disorders; (2) a history of substance use disorder, traumatic brain injury, or loss of consciousness; (3) intellectual disability or impaired cognitive functioning; and (4) comorbid psychiatric diagnoses other than major depressive disorder. Between December 2021 and January 2024, a total of 125 participants meeting the above criteria were recruited from inpatient and outpatient settings at the Fourth People’s Hospital of Chengdu.

### 2.2 Demographic and clinical measures

Demographic information including age, sex, educational attainment, living arrangements, and parental marital status was collected. Additionally, clinical data were obtained using standardized psychological assessments, as detailed below.

#### 2.2.1 Childhood trauma questionnaire-short form

To assess the severity of childhood trauma, the Chinese version of the CTQ-SF was utilized to evaluate the severity of child abuse and neglect that individuals may have encountered before the age of 16 (23). The CTQ-SF is a 28-item self-report questionnaire, including 25 clinical items and 3 validity items, for brief screening of maltreatment histories during childhood for both clinical and nonreferred individuals. Each item is scored ranging from 1 (never true) to 5 (very often true), and five dimensions of maltreatment histories are assessed which are emotional abuse, physical abuse, sexual abuse, emotional neglect, and physical neglect. Childhood trauma exposure was assessed using the criteria of emotional abuse≥13, physical abuse≥10, sexual abuse≥8, emotional neglect≥15, and physical neglect≥10. The Cronbach’s α coefficient for this study was 0.869.

#### 2.2.2 Beck scale for suicidal ideation

Klonsky et al. (24) have summarized that most clinical factors associated with suicidal behavior are primarily linked to suicidal ideation rather than suicide attempts, underscoring the critical role of suicidal ideation in suicide risk assessment. Therefore, this study utilized suicidal ideation to represent the suicide risk of the participants. Specifically, the Chinese version of the Beck Scale for Suicide Ideation (BSS) was employed to assess an individual’s current suicide risk. The BSS is a 19-item self-report instrument designed to quantify the intensity of conscious suicidal intent by measuring various dimensions of self-destructive thoughts or wishes (25). Each item consists of three alternative statements rated on a 3-point intensity scale from 0 to 2. Higher scores indicate greater current suicidal ideation and elevated suicide risk. The Cronbach’s α coefficient for this study was 0.941.

#### 2.2.3 Emotion regulation questionnaire

In this study, the Chinese version of the Emotion Regulation Questionnaire (ERQ) was used to assess participants’ habitual use of two key emotion regulation strategies: cognitive reappraisal and expressive suppression (14). This widely validated self-report measure comprises two independent subscales: a six-item Reappraisal scale and a four-item Suppression scale. Participants rated each item on a 7-point Likert scale ranging from 1 (strongly disagree) to 7 (strongly agree). Higher scores on each subscale indicate a greater tendency or more frequent use of the corresponding emotion regulation strategy. The Cronbach’s α coefficient of this scale in this study was 0.802.

### 2.3 Statistical analysis

Statistical analyses were performed using the SPSSPRO software V 1.0.11 (Online Application Software, China, https://www.spsspro.com). The descriptive statistics were used to present the demographic and clinical characteristics of the employed depressed patients with NSSI behavior. This included calculating the distribution of total scores for the CTQ-SF, its subscales representing different trauma subtypes, the Reappraisal scale, the Suppression scale, and the BSS. The prevalence of positive exposure for experiences of any subtype of childhood trauma and for each childhood trauma subtype was also computed. Furthermore, we analyzed the distribution of these clinical variables within different demographic subgroups and compared their differences between these subgroups. The Shapiro-Wilk test was used to assess data normality. For non-normally distributed data, non-parametric tests were applied: the Mann-Whitney U test for two-group comparisons and the Kruskal-Wallis H test for multi-group comparisons following *post-hoc* analyses. Relationships among the scores of each childhood trauma subtype, the Reappraisal scale, the Suppression scale, and the BSS were examined using Pearson correlation. Stepwise linear regressions were then conducted to identify which childhood trauma subtype(s) were significant predictors of the scores of the Reappraisal scale, the Suppression scale, and the BSS, respectively. For predictor(s) significant for both the Reappraisal scale and the BSS, or for both the Suppression scale and the BSS, mediation analyses were performed with 5,000 bootstrap samples to investigate the mediating role of the cognitive reappraisal and the expressive suppression between the childhood trauma predictor(s) and the BSS, respectively. A significant mediation effect was inferred if the 95% bias-corrected confidence interval (CI) for the path coefficient did not contain zero.

## 3 Results

### 3.1 Demographics

A total of 125 depressed adolescents and young adults accompanied with NSSI behavior were enrolled, among which 5 were excluded due to incomplete demographic information. The data of remaining 120 participants were used to investigate the mediation effects. The age of the participants ranged from 11 to 22 years with the mean age of ((14.700 ± 1.822) years and the mean years of schooling were (9.033 ± 1.829) years. We didn’t get any participants outside the 11–22 years range. Twenty-three of them were males and 97 were females.

### 3.2 Characteristics of childhood trauma, emotion regulation and suicidal ideation

According to the criteria for positive exposure to childhood trauma established by Bernstein et al. (23), 82.5% of the participants experienced at least one subtype of childhood trauma. Additionally, 55.8% of participants reported positive emotional abuse, 33.2% reported physical abuse, 10.8% reported sexual abuse, 68.3% reported emotional neglect, and 52.5% reported physical neglect. Detailed characteristics of positive trauma exposure are presented in **Table 1**.

**Table 1:** Positive exposure of childhood trauma in depressed adolescents and young adults with NSSI (n=120).

Variables	Minimum score	Maximum score	Mean score ± SD	Number of positive exposure (%)
CTQ total	25	94	54.43 ± 15.097	99(82.5%)
Emotional abuse	4	25	13.34 ± 5.035	67(55.8%)
Physical abuse	5	25	8.83 ± 4.304	40(33.2%)
Sexual abuse	3	21	5.79 ± 2.372	13(10.8%)
Emotional neglect	5	25	16.27 ± 5.094	82(68.3%)
Physical neglect	5	24	10.18 ± 4.087	63(52.5%)

NSSI, nonsuicidal self-injury; CTQ, Children Trauma Questionnaire; SD, standard deviation.

Differences across demographic subgroups were examined for all subtypes of childhood trauma, cognitive reappraisal, expressive suppression, and suicidal ideation. Specifically, total CTQ scores, emotional abuse subscale scores, and emotional neglect subscale scores differed significantly by parental marital status (*p* = 0.040 for total CTQ, *p* = 0.017 for emotional abuse, and *p* = 0.040 for emotional neglect). *Post-hoc* analyses indicated that participants with divorced parents reported significantly higher total CTQ scores, emotional abuse scores, and emotional neglect scores compared to those with married parents (all *post-hoc p*-values < 0.05). Additionally, female participants reported higher total CTQ scores than males (*p* = 0.036) and lower cognitive reappraisal scores (*p* = 0.050). No significant differences were observed among demographic subgroups for other trauma subtypes or the remaining clinical measures. Detailed results are presented in **Table 2**.

**Table 2:** Demographically associated clinical characteristics in depressed adolescents and young adults with NSSI (n=120).

Categories of demographic variables	Emotional abuse score (mean ± SD)	Physical abuse score (mean ± SD)	Sex abuse score (mean ± SD)	Emotional neglect score (mean ± SD)	Physical neglect score (mean ± SD)	CTQ total score (mean ± SD)	Reappraisal score (mean ± SD)	Suppression score (mean ± SD)	BSS score (mean ± SD)
*General* (n = 120)	13.34 ± 5.035	8.83 ± 4.304	5.79 ± 2.372	16.27 ± 5.094	10.18 ± 4.087	54.43 ± 15.097	21.15 ± 6.732	17.23 ± 5.300	14.93 ± 8.392
*Sex*	*P* = 0.256	*P* = 0.781	*P* = 0.073	*P* = 0.066	*P* = 0.099	*P* = 0.039	*P= 0.050*	*P= 0.321*	*P* = 0.345
Male (n =23)	12.39 ± 4.803	8.26 ± 3.414	5.30 ± 1.743	14.39 ± 5.876	8.83 ± 3.143	49.17 ± 14.559	23.78 ± 6.599	17.74 ± 6.054	13.35 ± 8.611
Female (n =97)	13.57 ± 5.087	8.97 ± 4.494	5.91 ± 2.492	16.71 ± 4.817	10.49 ± 4.231	55.67 ± 15.025	20.53 ± 6.644	17.10 ± 5.133	15.31 ± 8.341
*Period of age*	*P* = 0.588	*P* = 0.378	*P* = 0.568	*P* = 0.969	*P* = 0.675	*P* = 0.714	*P = 0.442*	*P = 0.764*	*P* = 0.200
Early/middle adolescence (10-14yrs) (n =81)	13.41 ± 4.56	8.85 ± 4.031	5.73 ± 2.393	16.33 ± 4.914	9.93 ± 3.563	54.27 ± 13.155	20.90 ± 6.614	17.38 ± 5.274	15.59 ± 7.767
Late adolescence and young adulthood (> 14ys) (n = 39)	13.21 ± 5.966	8.79 ± 4.878	5.92 ± 2.355	16.13 ± 5.512	10.69 ± 5.017	54.74 ± 18.688	21.67 ± 7.031	16.90 ± 5.409	13.56 ± 9.525
*Parents’ marital status*	*P* = 0.017	*P* = 0.506	*P* = 0.111	*P* = 0.040	*P* = 0.273	*P* = 0.040	*P = 0.730*	*P = 0.954*	*P* = 0.221
Married (n = 74)	12.27 ± 4.266	8.32 ± 3.735	5.31 ± 1.084	15.45 ± 5.099	9.69 ± 3.716	51.07 ± 11.855	21.18 ± 6.945	17.18 ± 5.321	14.05 ± 7.827
Divorced (n = 40)	14.88 ± 5.915	9.63 ± 5.037	6.78 ± 3.669	17.75 ± 4.908	11.18 ± 4.657	60.20 ± 18.402	20.88 ± 6.537	17.33 ± 5.526	16.80 ± 9.255
Other (n = 6)	16.33 ± 4.274	9.83 ± 5.419	5.17 ± 0.408	16.50 ± 4.722	9.50 ± 3.834	57.33 ± 16.955	22.67 ± 6.154	17.17 ± 4.119	13.33 ± 8.454
*Way of living*	*P* = 0.394	*P* = 0.986	*P* = 0.083	*P* = 0.828	*P* = 0.323	*P* = 0.544	*P= 0.648*	*P= 0.800*	*P* = 0.662
Living alone (n = 3)	8.67 ± 4.041	8.00 ± 0.000	5.00 ± 0.000	16.00 ± 4.359	10.67 ± 2.517	48.33 ± 6.506	25.00 ± 6.245	17.00 ± 2.646	9.00 ± 10.583
School dormitory (n = 18)	13.89 ± 5.411	9.00 ± 4.419	6.39 ± 2.570	17.17 ± 5.691	12.56 ± 5.963	59.00 ± 19.626	19.83 ± 6.964	15.94 ± 6.005	14.78 ± 8.558
Living with families (n = 98)	13.39 ± 4.990	8.84 ± 4.390	5.70 ± 2.378	16.11 ± 5.058	9.72 ± 3.586	53.78 ± 14.344	21.24 ± 6.740	17.48 ± 5.255	15.11 ± 8.369
Other (n = 1)	13.00	8.00	6.00	16.00	10.00	54.00	24.00	16.00	18.00

NSSI, nonsuicidal self-injury; SD, standard deviation; CTQ, Children Trauma Questionnaire; BSS, Beck Scale for Suicidal Ideation.

### 3.3 Correlations among childhood trauma, emotion regulation and suicidal ideation

The results of Pearson correlation analysis indicated several key findings. A significant negative correlation was observed between cognitive reappraisal and emotional neglect (*r* = -0.233, *p* < 0.01) among depressed adolescents and young adults with NSSI. However, no significant correlations were detected between expressive suppression and any childhood trauma subtypes. Additionally, current suicidal ideation showed significant positive correlations with emotional abuse (*r* = 0.316, *p* < 0.001), emotional neglect (*r* = 0.313, *p* < 0.001), and physical neglect (*r* = 0.300, *p* < 0.001), while a significant negative correlation was identified with cognitive reappraisal (*r* = -0.287, *p* < 0.01). Further details are provided in **Table 3**.

**Table 3:** Correlation matrix of main study constructs in depressed adolescents and young adults with NSSI (n=120).

Variables	1	2	3	4	5	6	7	8
1. Emotional abuse	1							
2. Physical abuse	0.465***	1						
3. Sexual abuse	0.299***	0.221*	1					
4. Emotional neglect	0.486***	0.296***	0.272**	1				
5. Physical neglect	0.436***	0.269**	0.311***	0.626***	1			
6. Reappraisal	-0.103	-0.090	-0.106	-0.233^**^	-0.141	1		
7. Suppression	0.177	0.016	0.050	-0.040	0.077	0.377^***^	1	
8. BSS	0.316***	0.167	0.091	0.313***	0.300^***^	-0.287**	0.072	1
Mean	13.34	8.83	5.79	16.27	10.17	21.15	17.23	14.93
SD	5.035	4.304	2.372	5.094	4.087	6.732	5.300	8.392

NSSI, nonsuicidal self-injury; BSS, Beck Scale for Suicidal Ideation; SD, standard deviation. *p < 0.05, **p < 0.01, ***p < 0.001.

### 3.4 Mediating effects of emotion regulation in the relationship between childhood trauma subtypes and suicidal ideation

Since no significant correlations were found between expressive suppression and any childhood trauma subtypes, it was excluded from further mediation analysis. Consequently, we proceeded to examine the mediating role of cognitive reappraisal in the relationship between childhood trauma subtypes and current suicidal ideation.

In the initial step of stepwise linear regression analyses, we used the Reappraisal scores and the BSS scores (measuring suicidal ideation) as the dependent variables respectively, and the five childhood trauma subtypes—emotional abuse, physical abuse, sex abuse, emotional neglect, and physical neglect— as independent variables, to identify significant predictors. The results indicated that emotional neglect was the only significant predictor for the Reappraisal scale scores, and emotional abuse and emotional neglect were found to be significant predictors for the BSS scores; the other predictors of trauma subtypes were identified.

Since emotional neglect were the only significant predictor for both the Reappraisal scale scores and the BSS scores, mediating effects were subsequently investigated using Reappraisal scores as mediating variables between emotional neglect and BSS. In the first step (Model 1), emotional neglect had a significant influence on BSS (B = 0.515, *p* < 0.001), indicating that there existed a total effect. In the second step (Model 2), emotional neglect had a significant influence on Reappraisal scale scores (B = -0.308, *p* < 0.01). In Model 3, emotional neglect and Reappraisal both had significant effects on BSS (B = 0.429, *p* < 0.01; B = -0.282, *p* < 0.05). Therefore, the effect of emotional neglect on the BSS scores was partially mediated by the Reappraisal scale scores (for details, see **Table 4**). The significance of mediation effects was validated through bootstrap method. The indirect effect value was 0.087, and the 95% CI [0.006 - 0.234] did not contain 0 which indicates an indirect effect in the above models. The effect proportion of the Reappraisal scale scores on emotional neglect was 16.9%. Details were presented in **Table 5**.

**Table 4:** The mediating role of reappraisal in the relationship between emotional neglect and BSS.

Model		Model 1		Model 2		Model 3
Dependent variable		BSS		Reappraisal		BSS
Indicator	B	t	B	t	B	t
Emotional neglect	0.515	3.577^***^	-0.308	-2.603^**^	0.429	2.961^**^
Reappraisal					-0.282	-2.572^*^
R^2^	0.098		0.054		0.146	
F	12.796		6.774		10.011	

BSS, Beck Scale for Suicidal Ideation. *p < 0.05, **p < 0.01, ***p < 0.001.

**Table 5:** The mediation effects of reappraisal using bootstrap methods for validation between emotional neglect and BSS.

Mediation path	Total effect value	Indirect effect value	Indirect effect, 95% bootstrap confidence interval	Proportion mediated %
Emotional neglect=>Cognitive reappraisal=>BSS	0.515	0.087	0.006 - 0.234	16.90%

BSS, Beck Scale for Suicidal Ideation.

## 4 Discussion

This study elucidates the complex interrelationships between specific subtypes of childhood trauma, core emotion regulation strategies, and current suicidal ideation within a high-risk clinical population: depressed adolescents and young adults with NSSI. Our findings delineate a distinct clinical profile characterized by a high prevalence of emotional forms of maltreatment and provide evidence for a specific psychological mechanism linking early adversity to suicide risk, advancing our mechanistic understanding of suicidality in this comorbid population.

### 4.1 Predominance of emotional maltreatment in depressed youth with NSSI

A primary finding is the distinct distribution of childhood trauma subtypes in our sample. Emotional neglect (68.3%), emotional abuse (55.8%), and physical neglect (52.5%) were the most prevalent forms, affecting the majority of participants. This profile diverges significantly from patterns observed in general population surveys. For instance, the WHO World Mental Health Surveys across diverse nations reported notably lower prevalence rates, with physical abuse (10.8%) being more common than neglect (5.2%) and sexual abuse (0.6%) being the least reported (26). The substantially elevated rates of all trauma subtypes in our sample underscore the profound burden of early adversity in this comorbid clinical group. This pattern aligns with prior research on Chinese adolescents and university students with NSSI (27, 28), suggesting a potential socio-cultural or clinical specificity. It is noteworthy that while a global integrative study linked NSSI to all forms of maltreatment except emotional neglect (29), and a meta-analysis emphasized emotional/sexual abuse as key risk factors for NSSI (30), our data position emotional neglect and abuse as the predominant trauma types in youth with co-occurring depression and NSSI. This highlights the critical, yet often overlooked, impact of emotional neglect—a form of maltreatment that research in high-income countries has shown to exert long-term mental health consequences comparable to, or even exceeding, those of physical or sexual abuse (31). This discrepancy also points to the heterogeneity within NSSI populations, where comorbid depression may be associated with a distinct trauma etiology centered on emotional deprivation and invalidation. We acknowledge that our sample was recruited from an affluent urban center in China; future research must investigate whether this trauma profile holds in less developed regions to understand potential geographical and socioeconomic moderators.

Additionally, our study found that participants with divorced parents reported significantly higher levels of childhood trauma, particularly emotional abuse and emotional neglect, compared to those with married parents. These results suggest that parental divorce may heighten the risk of exposure to emotional neglect and abuse during childhood. While few studies have directly compared the prevalence of childhood trauma between offspring of divorced versus married parents, prior research indicates that parental separation is associated with an elevated risk of developing psychotic disorders (32). Furthermore, our data indicate that female participants may experience more childhood trauma than males. This contrasts with a study from Pakistan, which reported a higher distribution of childhood abuse and neglect among males (33). However, another line of evidence suggests that the impact of childhood trauma on psychotic disorders, such as psychosis, may be more pronounced in females, manifesting as more severe symptoms and functional impairment (34). This could partially explain why, in our clinical sample, females appeared to report greater exposure to traumatic childhood experiences.

### 4.2 Subtype-specific links between childhood trauma and suicidal ideation

A central finding is the delineation of specific childhood trauma subtypes associated with suicidal ideation. Elevated levels of emotional abuse, emotional neglect, and physical neglect were all significantly associated with increased current suicidal ideation, with emotional neglect and abuse emerging as particularly salient predictors. This extends the robust literature establishing childhood trauma as a transdiagnostic risk factor for suicidal thoughts and behaviors (35–38), independently of comorbid anxiety and depression (39). To our knowledge, this is the first study to examine these subtype-specific associations within the high-risk, comorbid population of depressed adolescents and young adults with NSSI.

The non-significant associations for sexual abuse and physical abuse with suicidal ideation warrant careful interpretation. This aligns with research suggesting sexual abuse may have a stronger link to suicide attempts than to ideation (40, 41), and that certain forms of physical trauma may not directly correlate with ideation (42). The inclusion of both sexes in our sample may have attenuated a potentially stronger female-specific association between sexual abuse and ideation reported before (43). However, discrepancies exist across populations, underscoring the context-dependent nature of these links. For example, physical neglect is a strong risk factor for suicidal ideation in patients with schizophrenia (44) but not in Chinese college students (45). Similarly, childhood sexual abuse directly predicts suicidal ideation in some adult clinical samples (9, 46). These variations highlight that the impact of specific trauma subtypes on suicide risk exhibits both transdiagnostic relevance and population-specific variations, likely moderated by factors such as diagnosis, developmental stage, and cultural context. Our findings emphasize that for youth with depression and NSSI, emotional forms of maltreatment are critically implicated in suicidal thinking.

### 4.3 Emotion regulation as a specific mediating pathway

The study’s most significant advancement lies in identifying a specific psychological mechanism. We found that childhood emotional neglect uniquely predicted decreased use of cognitive reappraisal, a key adaptive emotion regulation strategy. This aligns with and extends evidence from depressed adults and college students showing similar associations (47, 48). This specificity suggests that emotional neglect—characterized by a failure to meet emotional needs and psychological needs (23)—may fundamentally impair the development of the cognitive capacity to reframe negative experiences, a skill essential for resilience.

In contrast, no trauma subtype predicted the use of expressive suppression, a finding consistent with reports in borderline personality disorder and pediatric depression (49, 50), though it contrasts with other studies focusing on adolescent anxiety, obsession, and adult depressive symptoms (16, 17, 48). Given that NSSI behaviors are commonly observed in individuals with borderline personality disorder and that our sample predominantly consisted of young patients with depression, our results align with findings from these specific populations, suggesting that childhood trauma may not be strongly associated with expressive suppression in such clinical groups. Therefore, we hypothesize that the clinical characteristics—particularly the age and diagnostic background of the participants—may account for these discrepancies across studies.

Crucially, cognitive reappraisal was found to partially mediate the relationship between emotional neglect and suicidal ideation, accounting for 16.9% of the total effect. This mediation model is coherent with prior research establishing cognitive reappraisal as a mediator between childhood trauma and various forms of psychopathology, including anxiety, depression, and PTSD (16, 51–53). A pivotal longitudinal study also found that access to emotion regulation strategies mediated the childhood maltreatment-suicidal ideation link in psychiatrically hospitalized adolescents (19). Our study advances this literature by pinpointing cognitive reappraisal as the specific mediator in the pathway from a specific trauma subtype (emotional neglect) to suicidal ideation within a well-defined, high-risk clinical population. No other trauma subtype operated through this pathway in our model, highlighting the precision of this mechanism.

Neurobiologically, this mediation finds plausible support in models of frontal-amygdala circuitry. Childhood trauma is known to alter functional activity and connectivity in the prefrontal cortex and amygdala (52), regions that are central to the cognitive control and reappraisal of emotional stimuli (54). Importantly, dysfunction within these same neural circuits is consistently documented in individuals at high risk for suicide (55, 56), providing a convergent biological substrate for our psychological findings. Thus, the pathway from emotional neglect to impaired reappraisal and subsequently to suicidal ideation is not only statistically supported but also conceptually coherent across psychological and neurobiological levels of analysis.

## 5 Limitations

Several limitations warrant consideration. First, the cross-sectional design precludes causal inference regarding the mediation pathway. Second, reliance on retrospective self-report measures may introduce recall and social desirability biases. Third, the present study did not account for the severity of NSSI, such as its frequency, which may introduce heterogeneity in NSSI characteristics across participants and potentially influence the results of the mediation analysis. Future studies with larger sample sizes are needed to more thoroughly investigate how emotion regulation functions mediate the relationship between childhood trauma and suicide risk across different NSSI subtypes. Fourth, the generalizability of findings may be constrained by the small to modest sample size and its origin from a single urban center. Fifth, the observed mediation effect of cognitive reappraisal was small-to-moderate, indicating that other significant mediators (e.g., other emotion regulation strategies, interpersonal factors, neurobiological changes) likely exist and require exploration in future research. Finally, the absence of clinical control groups (e.g., depressed youth without NSSI) limits our ability to attribute findings specifically to the NSSI component of the comorbidity.

## 6 Conclusion and implications

This study provides a nuanced model for understanding suicide risk in depressed adolescents and young adults with NSSI. We identify a high-prevalence trauma profile centered on emotional maltreatment and demonstrate that childhood emotional neglect contributes to suicidal ideation, partially through the specific mechanism of impairing cognitive reappraisal. These findings have clear clinical implications. They advocate for the routine, detailed, and subtype-specific assessment of childhood trauma—with particular attention to emotional neglect and abuse—in the clinical management of depressed youth with NSSI. Furthermore, cognitive reappraisal is highlighted as a promising and specific intervention target. Therapeutic approaches such as Cognitive Behavioral Therapy (CBT) and Dialectical Behavior Therapy (DBT), particularly modules designed to enhance cognitive reappraisal skills, could be tailored for this population. Strengthening this capacity may help buffer the long-term impact of emotional neglect and mitigate its pathway to suicidal thinking.

However, it should be noted that in the present study, cognitive reappraisal accounted for less than one-fifth of the total effect of emotional neglect on current suicide risk in depressed adolescents and young adults with NSSI. We speculate that other factors may also mediate the relationship between emotional neglect and suicide risk in this population, such as impulsivity, hopelessness, social support, and self-esteem, which have been previously linked to suicidality in adolescents (24, 57). Future research on suicide risk in individuals with NSSI should further and more thoroughly explore the impact of multidimensional psychosocial factors.

Despite its limitations, this study establishes a foundational model linking specific childhood trauma subtypes to suicidality via a defined emotion regulation pathway. It offers a potential direction for future longitudinal and neurobiological research to confirm causality and for developing targeted, mechanism-informed interventions for this vulnerable population.

## Data Availability

The raw data supporting the conclusions of this article will be made available by the authors, without undue reservation.
